# Epicardial Adipose Tissue Measured From Computed Tomography Predicts Cardiac Resynchronization Therapy Response in Patients With Non-ischemic Systolic Heart Failure

**DOI:** 10.3389/fcvm.2021.678467

**Published:** 2021-10-28

**Authors:** Hui-yuan Qin, Cheng Wang, Duo-duo Qian, Chang Cui, Ming-long Chen

**Affiliations:** ^1^Department of Cardiology, The First Affiliated Hospital of Nanjing Medical University, Nanjing, China; ^2^Department of Cardiology, The Affiliated Jiangning Hospital of Nanjing Medical University, Nanjing, China

**Keywords:** epicardial adipose tissue (EAT), cardiac CT, cardiac resynchronization therapy (CRT), heart failure (HF), single photon emission computed tomography (SPECT)

## Abstract

**Background:** Epicardial adipose tissue (EAT) has been linked with the pathogenesis of heart failure (HF). Limited data have been reported about the clinical value of EAT for cardiac resynchronization therapy (CRT) in non-ischemic systolic HF. We aimed to explore the values of EAT measured from CT to predict the response to CRT in patients with non-ischemic systolic HF.

**Methods:** Forty-one patients with CRT were consecutively recruited for our study. All patients received both gated resting Single Photon Emission CT (SPECT) myocardial perfusion imaging (MPI) and dual-source multi-detector row CT scans. EAT thickness was assessed on both the parasternal short and horizontal long-axis views. The area of EAT was calculated at the left main coronary artery level. Left ventricular systolic mechanical dyssynchrony (LVMD) was measured by phase standard deviation (PSD) and phase histogram bandwidth (PBW). The definition of CRT response was an improvement of 5% in left ventricular ejection fraction (LVEF) at 6 months after CRT implantation.

**Results:** After 6 months of follow-up, 58.5% (24 of 41) of patients responded to CRT. A greater total perfusion deficit (TPD) was observed in the left ventricle, and a narrower QRS complex was observed in the nonresponse group than in the response group (*p* < 0.05). Meanwhile, the systolic PSD and systolic PBW were statistically greater in the CRT group with no response than in the response group (*p* < 0.05). Meanwhile, the baseline QRS duration, TPD, systolic PSD, systolic PBW, EAT thicknesses of the left ventricular (LV) apex, right atrioventricular (AV) groove, and left AV groove were all significantly related to the CRT response in the univariate logistic regression analysis. Furthermore, the QRS duration and EAT thicknesses of the right AV groove and left AV groove were independent predictors of CRT response in the multivariate logistic regression analysis.

**Conclusions:** The EAT thickness of the left AV groove in patients with non-ischemic systolic HF is associated with the TPD of LV and LV systolic dyssynchrony. The EAT thickness of the AV groove has a good predictive value for the CRT response in patients with non-ischemic systolic HF.

## Introduction

Cardiac resynchronization therapy (CRT) is an effective option for patients with medically refractory heart failure (HF). However, one-third of patients with HF treated with CRT have a suboptimal response in clinical practice (1). As the implantation of CRT devices is associated with the risk of device implantation, periprocedural complications, and relatively high costs, optimizing the current selection of patients with CRT is essential. Research in this area has shown that the CRT response is influenced greatly by left ventricular (LV) myocardial tissue viability, left ventricular dyssynchrony (LVMD), lead implantation, and fibrosis (2). Our previous study (3) also found that higher scar burden and LV lead implantation in scarred areas were associated with a suboptimal CRT response in patients with non-ischemic systolic HF.

Epicardial adipose tissue (EAT) is speculated to be linked with microvascular dysfunction, impairment of functional myocardium, and cardiac fibrosis independent of traditional risk factors (4). Previous studies (5, 6) have found that EAT is a novel parameter for cardiovascular risk assessment in coronary artery disease (CAD), cardiac hypertrophy, and atrial fibrillation (AF). Moreover, a recent study also found that EAT, which plays a role as electrical insulation, could potentially interfere with sensing and pacing for lead design in CRT (7). Nevertheless, performing LV lead placement and electrode positioning according to EAT may lead to uncertainty.

However, little data have been obtained about the clinical value of EAT for CRT in patients with non-ischemic systolic HF. We aimed to explore the values of EAT measured from CT to predict the CRT response in patients with non-ischemic systolic HF.

## Methods

### Patient Population

Forty-one patients with CRT with non-ischemic systolic HF (8) were consecutively recruited for our research from October 2016 to August 2020 at the First Affiliated Hospital of Nanjing Medical University. Patients enrolled in our study received both resting Single Photon Emission Computed Tomography (SPECT) myocardial perfusion imaging (MPI) and dual-source multi-detector row CT scans. In our study, the CRT indications were as follows: (1) sinus rhythm; (2) left ventricular ejection fraction (LVEF) ≤35%; (3) New York Heart Association (NYHA) functional class from II to IV; (4) completed left bundle branch block (LBBB) morphology; and (5) optimal medical treatment for HF at least 3 months before implantation. Individuals who had AF, right bundle branch block, or right ventricular pacing upgradation were excluded. Coronary artery disease (CAD) was excluded by heart CT scan, and all patients had stenosis of <50% in the epicardial coronary artery. This study protocol was approved by the Regional Ethics Committee (the First Affiliated Hospital of Nanjing Medical University), and written informed consent was obtained for enrolled patients.

### Electrocardiography and Echocardiography

An electrocardiogram (ECG) was acquired by researchers during hospitalization. The QRS duration in 12-lead ECG was analyzed from the widest QRS complex.

Left ventricular diameter parameters and diastolic function were measured by two experienced imaging-specialized experts according to standard transthoracic echocardiography. LV parameters included left ventricular end-diastolic dimension (LVEDD), left ventricular end-systolic dimension (LVESD), and LVEF. The diastolic function included mitral inflow E velocity to tissue Doppler e' velocity ratio (E/e') and mitral inflow E velocity to mitral inflow A velocity ratio (E/A). Both experts were blinded to the other clinical data during the research. The biplane-modified Simpson method was used to record the LVEF.

### Gated Myocardial Perfusion SPECT

The resting ECG-gated SPECT MPI scan was completed in patients before implantation. Approximately 20–30 mCi of Tc-99m sestamibi was injected, and the MPI scan was continued 60 min after injection. The MPI images were assessed by a dual-headed camera using a routine protocol (CardioMD, Philips Medical Systems, Amsterdam, Netherlands). Reconstruction and reorientation to MPI images were performed using the Emory Toolbox (Syntermed, Atlanta, GA, USA).

The resulting short-axis MPI images were performed to evaluate the LV contour parameters by inputting them into an interactive tool. These results in each cardiac frame were then put into an automatic myocardial sampling algorithm for maximal count circumferential profiles. Subsequently, the onset contraction of the left ventricle was acquired from a first-harmonic Fourier approximation (9). Global LV was measured according to the phase standard deviation (PSD) and phase histogram bandwidth (PBW) from the phase analysis. The mechanical dyssynchrony value (two SDs above the mean) was defined as systolic PSD = 36.5° and systolic PBW = 159.6°.

### CT Scan

**Figure 1:**
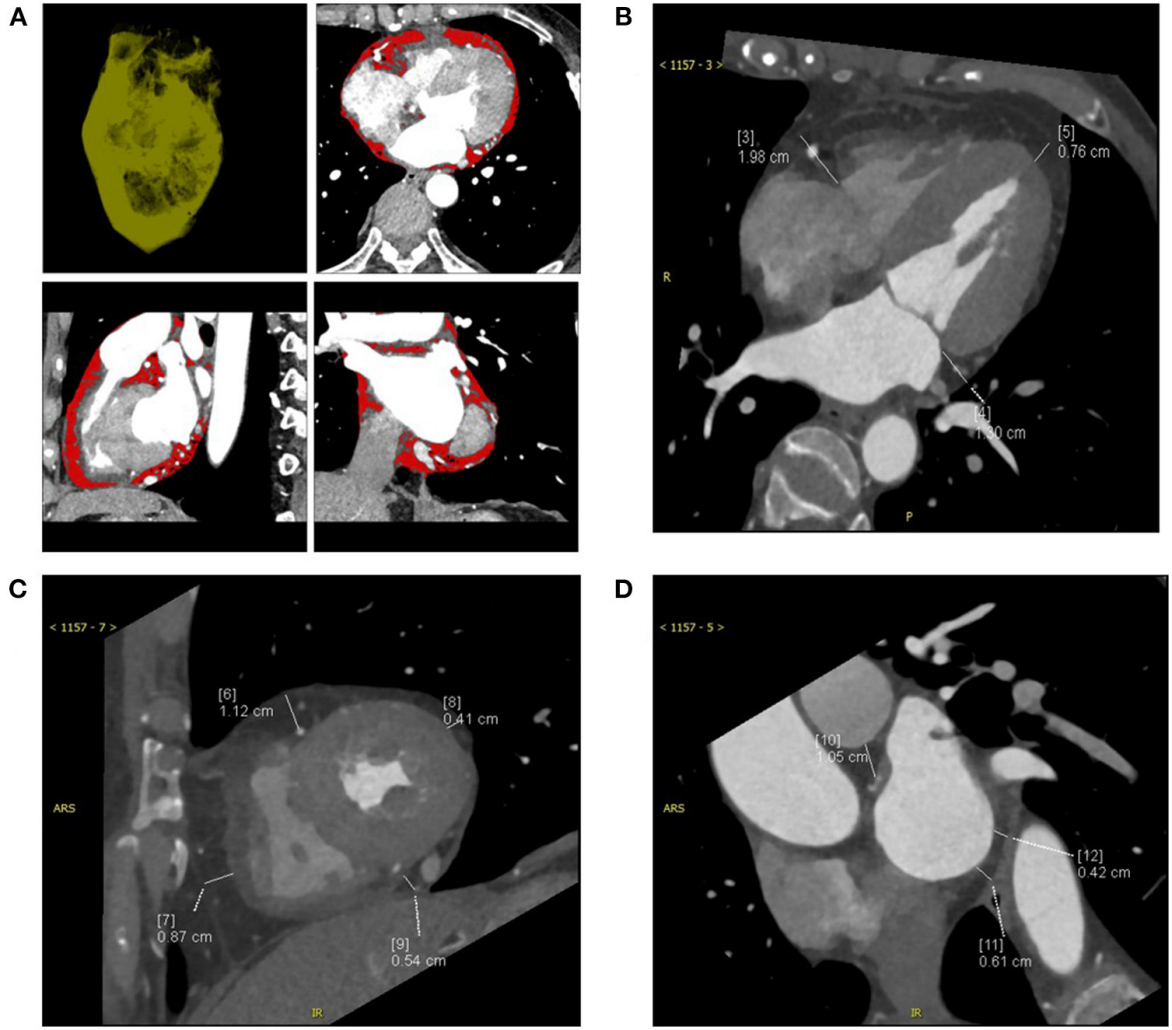
The computed tomography (CT) measurements of a patient with CRT-responsive participating in this study were shown and epicardial adipose tissue (EAT) was measured by multidetector CT. **(A)** Contrast-enhanced CT scan presenting the region of interest (ROI) and pixels attributed to epicardial adipose tissue (red areas). **(B)** EAT thickness was measured on the parasternal short-axis view. **(C)** EAT thickness was measured on the horizontal long-axis view. **(D)** EAT area was measured at the left main coronary artery level. CRT, cardiac resynchronization therapy.

All heart CT scans were acquired from a dual-source multi-detector-row scanner (Somatom Definition, Siemens Medical Solutions, Forchheim, Germany). CT scan data were as follows: detector collimation, 64 × 0.6 mm; gantry rotation time, 330 ms; pitch, 0.2–0.43 (adapted to the heartbeat); temporal resolution, 83 ms; tube current, 380–420 mAs per rotation; and tube potential, 100–120 kV depending on the body mass index (BMI). To minimize motion artifacts, all patients were requested to hold their breath during detection. We synchronized the data reconstruction by a retrospective gating technique according to the ECG signal in all cardiac phases at 40% of the left atrial volume max at a total slice thickness of 0.75 mm and a reconstruction increment of 0.4 mm. All EAT data were measured by one experienced radiologist, and the best diastolic phase images of all the patients were selected for analysis. The adipose tissue between the surface of the myocardium and epicardium was defined as EAT, and the EAT volume was automatically acquired by applying cardiac risk assessment software to the cardiac image (10) (Figure 1A). The EAT thickness in grooved segments was calculated in the horizontal long-axis plane (right atrioventricular (AV) groove and left AV groove), and the apical EAT thickness was also calculated using this image (10) (Figure 1B). The mean EAT thicknesses of the right ventricle (RV), lateral wall, and anterior wall were measured at two points on a parasternal short-axis view along the right ventricular (RV) free wall (Figure 1C). The EAT thickness over the lateral wall of the LV and posterior wall of the ventricle was also obtained on the parasternal short-axis view (11). The mean EAT thickness was calculated according to the lateral and posterior walls of the ventricle myocardial surface to the pericardium (Figure 1D).

### CRT Implantation

All enrolled individuals underwent CRT in standard procedures. The LV lead was steadily placed in one branch vein, and the right ventricular (RV) lead was positioned in the RV apex as determined by trained electrophysiologists following standard implantation guidelines. The final position was determined based on three factors: no phrenic nerve capture, good stability, and an acceptable pacing threshold. Finally, fluoroscopic venograms were used to assess the LV lead position implantation in the left anterior oblique 45° and right anterior oblique 30° regions.

### Follow-Up After CRT

Follow-up data of all the patients were collected from telephone interviews, hospital discharge summaries, and government records. The primary end point was the CRT response at 6 months after CRT implantation, which was defined as an improvement of 5% in the LVEF. All-cause mortality was the secondary end point in the whole study. Atrial arrhythmias and ventricular pacing rate during follow-up were detected and extracted from the device system. According to the follow-up data, patients were divided into two groups: the CRT response group and the CRT non-response group.

### Statistical Analysis

Data were analyzed with SPSS 21 (IBM, Chicago, IL, USA). Categorical variables were summarized as counts or percentages. Continuous variables are presented as the mean ± SD. The Pearson linear correlation coefficient was used to determine whether two variables were linearly related. Baseline clinical variables associated with the CRT response were used in univariate and multivariate logistic regression analyses. All variables with *p* < 0.10 in the univariate logistic regression analysis were included in the multivariate logistic regression analysis. A receiver operating characteristic (ROC) curve analysis was performed for the EAT variables to predict the probability value for CRT. A Kaplan-Meier analysis was performed between two groups to compare the all-cause mortality by a log-rank test. A two-sided *p* < 0.05 was considered statistically significant.

## Results

### Baseline Characteristics

Table 1 displays the baseline characteristics for 41 consecutive patients (31 male, 64.7 ± 10.5 years old). Differences were not observed in medical therapy before implantation between the two groups, and these interventions included diuretics (90.2%), an aldosterone blocker (90.2%), beta-blockers (90.2%), sacubitril/valsartan (43.9%), angiotensin II antagonists, or angiotensin-converting enzyme inhibitors (44.5%). The clinical characteristics were similar between the two groups in terms of sex, age, diabetes, hypertension, history of the previous hospitalization, NYHA class, BMI, N-terminal pro-natriuretic brain natriuretic peptide (NT-proBNP), LVEDD, LVESD, E/e', E/A, and LVEF (*p* > 0.05). However, in the non-response group, there was a narrower QRS duration and more total perfusion deficit (TPD) in the left ventricle than in the response group (*p* < 0.05). Meanwhile, the systolic PSD and systolic PBW were statistically greater in the CRT group with no response than in the response group (*p* < 0.05, for both).

**Table 1:** Baseline characteristics and left ventricular (LV) parameters of the enrolled patients.

**Baseline characteristic**	**All** **(*n* = 41)**	**Non-responders** **(*n* = 17)**	**Responders** **(*n* = 24)**	***p-*value**
Age (years)	64.7 ± 10.5	65.1 ± 12.2	64.5 ± 9.4	0.860
Male (*n*, %)	31 (75.6%)	13 (76.5%)	18 (75.0%)	0.915
Hypertension	30 (73.2%)	14 (41.1%)	16 (32.0%)	0.129
Diabetes	15 (36.6%)	7 (21.0%)	8 (23.5%)	0.756
BMI (kg/m^2^)	25.1 ± 2.5	25.3 ± 2.6	25.0 ± 2.4	0.672
QRS duration (ms)	171.1 ± 19.6	162.4 ± 24.6	177.3 ± 12.2	0.014
NT-proBNP	3916.64 ± 3385.6	4337.0 ± 3063.8	3618.9 ± 3630.7	0.510
History of previous hospitalization (*n*)	1.3 ± 1.6	1.8 ± 2.0	1.0 ± 1.1	0.344
NYHA class II/III/IV	21/13/7	8/7/2	13/6/5	0.931
**Medication**				
Sacubitril/valsartan	18 (43.9%)	8 (47.1%)	10 (41.7%)	0.110
ACE inhibitors/ARBs	17 (44.5%)	5 (29.4%)	12 (55.0%)	0.193
β-blockers	37 (90.2%)	14 (82.4%)	23 (95.8%)	0.157
Diuretics	37 (90.2%)	16 (94.1%)	21 (87.5%)	0.487
Aldosterone blocker	37 (90.2%)	16 (94.1%)	21 (87.5%)	0.487
**LV parameters**				
LVEDD (mm)	68.7 ± 7.6	70.9 ± 6.6	67.0 ± 8.0	0.108
LVESD (mm)	58.3 ± 7.9	60.7 ± 6.6	56.6 ± 8.3	0.102
E/e'	16.1 ± 6.5	15.7 ± 5.0	16.4 ± 7.4	0.743
E/A	1.2 ± 0.7	1.4 ± 1.0	1.0 ± 0.5	0.089
LVEF (%)	29.0 ± 8.5	30.8 ± 10.8	27.7 ± 6.4	0.268
Scar burden	29.4 ± 12.0	33.2 ± 14.0	26.8 ± 9.8	0.090
TPD	12.5 ± 9.7	16.2 ± 12.8	10.0 ± 5.8	0.042
Systolic PSD (°)	37.3 ± 18.3	44.4 ± 19.5	32.3 ± 15.8	0.034
Systolic PBW (°)	141.3 ± 79.8	174.6 ± 89.7	117.7 ± 63.8	0.022
**EAT measurements**				
Total volume EAT (ml)	101.2 ± 49.3	104.8 ± 55.0	98.7 ± 45.8	0.699
Anterior wall (mm)	0.4 ± 0.2	0.4 ± 0.2	0.4 ± 0.2	0.966
Inferior wall (mm)	0.2 ± 0.1	0.2 ± 0.1	0.2 ± 0.1	0.946
Left free wall (mm)	0.2 ± 0.2	0.3 ± 0.1	0.2 ± 0.2	0.420
Right free wall (mm)	0.5 ± 0.2	0.5 ± 0.2	0.4 ± 0.2	0.109
Ventricle apex (mm)	0.4 ± 0.2	0.5 ± 0.2	0.3 ± 0.2	0.039
Right AV groove (mm)	1.6 ± 0.5	1.8 ± 0.5	1.5 ± 0.4	0.022
Left AV groove (mm)	1.2 ± 0.3	1.4 ± 0.3	1.1 ± 0.1	0.020

*Data are expressed as the mean ± SD or number (percentage); BMI, body mass index; NT-proBNP, N-terminal pro-natriuretic brain natriuretic peptide; ACE, angiotensin-converting enzyme; ARBs, angiotensin II receptor blocker; LVEDD, left ventricular end-diastolic dimension; LVESD, left ventricular end-systolic dimension; E/e', mitral inflow E velocity to tissue Doppler e' velocity ratio; E/A, mitral inflow E velocity to mitral inflow A velocity ratio, LVEF, left ventricular ejection fraction; TPD, total perfusion deficit; PSD, phase standard deviation; PBW, phase histogram bandwidth; AV, atrial ventricular; EAT, epicardial adipose tissue*.

**Figure 2:**
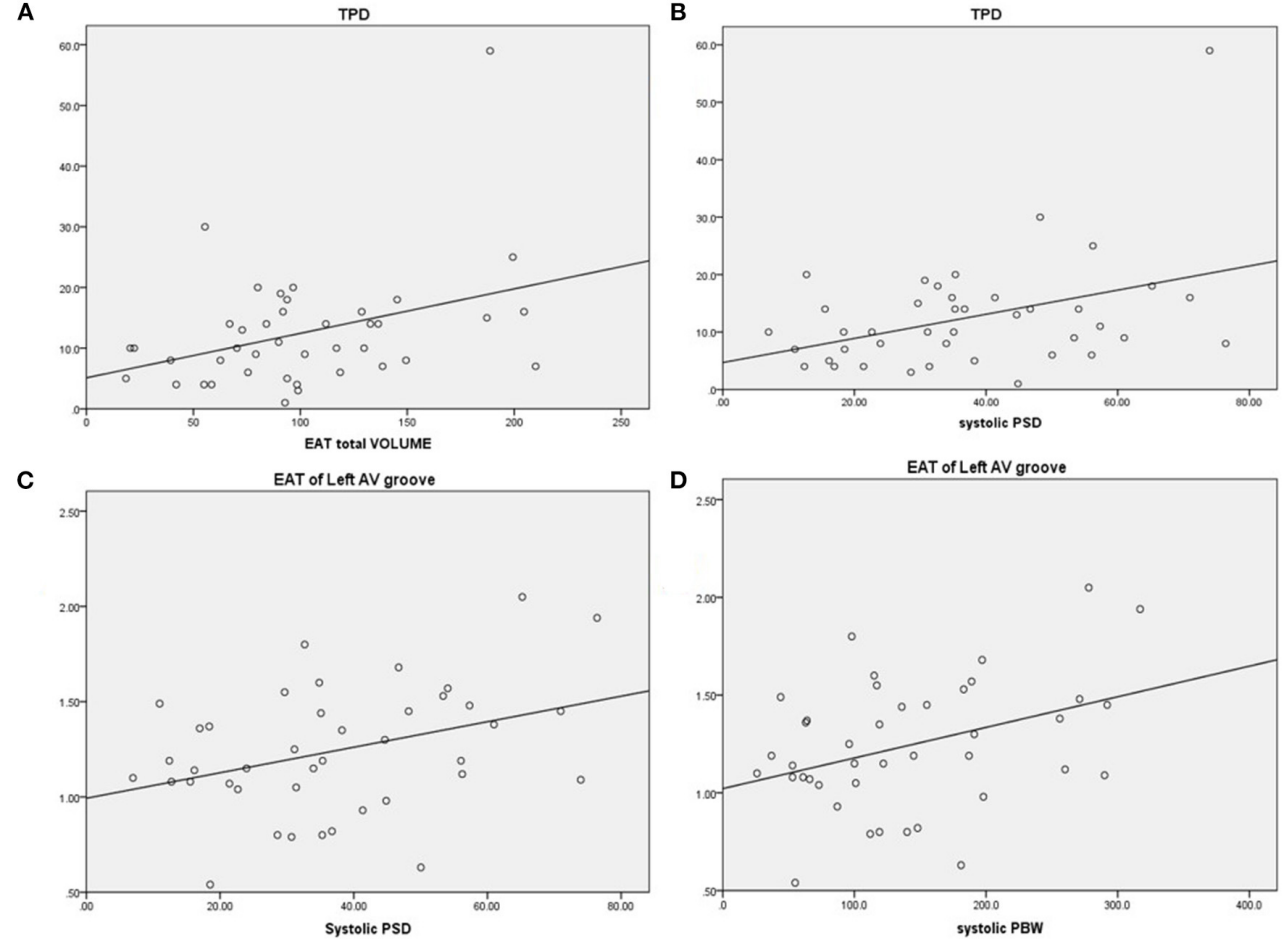
Correlations between EAT and LV parameters. **(A)** Total perfusion deficit (TPD) was correlated with greater EAT, *r* = 0.371, *p* = 0.017. **(B)** TPD was correlated with greater systolic PSD, *r* = 0.395, *p* = 0.011. **(C)** EAT of the left AV groove was correlated with greater systolic PSD, *r* = 0.367, *p* = 0.018. **(D)** EAT of the left AV groove was correlated with greater systolic PBW, *r* = 0.376, *p* = 0.016. EAT, epicardial adipose tissue; AV, atrioventricular; PSD, phase standard deviation; PBW, phase histogram bandwidth.

In addition to the total EAT thickness values, the EAT thickness over the LV lateral wall, LV anterior wall, LV inferior wall, and the right lateral wall did not differ between subjects with and without a CRT response (*p* > 0.05 for all). Notably, EAT thickness values in the right AV groove, LV apex, and left AV groove were lower in the CRT response group than the CRT group with no response (*p* < 0.05, for all). Figure 2 shows the relationship between the EAT volume and the LV parameters in bivariate correlation analysis. The total EAT volume was significantly correlated with the TPD levels (*r* = 0.371, *p* = 0.017). Meanwhile, a positive relationship was observed between the TPD and systolic SD levels (*r* = 0.395, *p* = 0.011). The EAT thickness of the left AV groove was positively correlated with systolic SD and systolic BW values (*r* = 0.367, *p* = 0.018; *r* = 0.376, *p* = 0.016, respectively).

### Prediction of CRT Response

**Figure 3:**
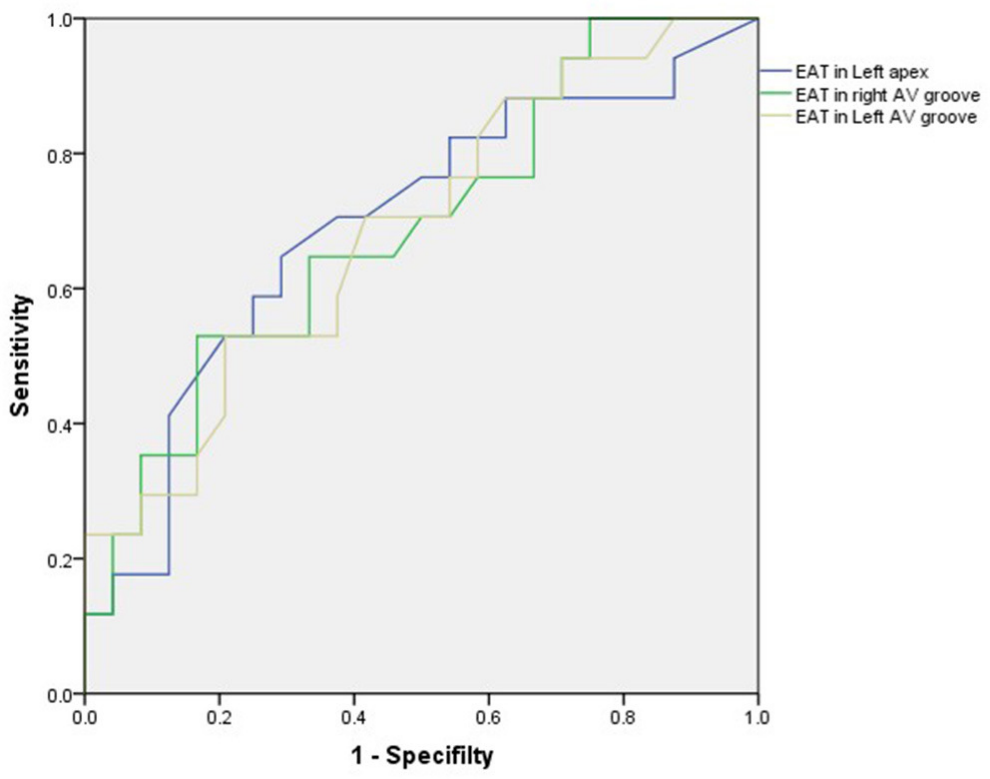
In the ROC analysis of the EAT thickness of the right AV groove, the area under the ROC curve (AUC), optimal cut off value, specificity, and sensitivity were 0.691, 1.84, 0.833, and 0.529, respectively (*p* = 0.039); meanwhile, in the ROC analysis of the EAT thickness of the left AV groove, the AUC, optimal cut off value, specificity and sensitivity were 0.687, 1.375, 0.792, and 0.529, respectively (*p* = 0.043). In the ROC analysis of the EAT thickness in the LV apex, the AUC, optimal cut off value, specificity, and sensitivity were 0.695, 0.415, 0.708, and 0.647, respectively (*p* = 0.035). AV, atrioventricular; EAT, epicardial adipose tissue; LV, left ventricular.

In total, 24 patients (58.5%) were CRT responders. LV leads were implanted in lateral, anterolateral, posterior, or posterolateral coronary veins according to the anatomic characteristics of coronary veins. In the univariate logistic regression analysis, the CRT response was significantly associated with the TPD, systolic PSD, systolic PBW, QRS duration, EAT thicknesses of LV apex, right AV groove, and left AV groove (Table 2). When multivariate logistic regression models were performed to test the EAT thickness parameters (LV apex, right AV groove, and left AV groove) separately, the right AV groove, left AV groove, and the QRS duration were independent predictors for the CRT response (Table 3). In the ROC analysis of the EAT thickness of the right AV groove, the area under the ROC curve (AUC), optimal cut off value, specificity, and sensitivity were 0.691, 1.84, 0.833, and 0.529, respectively (*p* = 0.039); meanwhile, in the ROC analysis of the EAT thickness of the left AV groove, the AUC, optimal cut off value, specificity, and sensitivity were 0.687, 1.375, 0.792, and 0.529, respectively (*p* = 0.043). According to the ROC analysis of the EAT thickness of the LV apex, the AUC, optimal cut off value, specificity, and sensitivity were 0.695, 0.415, 0.708, and 0.647, respectively (*p* = 0.035; Figure 3).

**Table 2:** Univariate logistic regression models for cardiac resynchronization therapy (CRT) response.

**Variables**	***P*-value**	**Hazard Ratio**	**95% CI**
QRS duration	0.028	1.050	1.005–1.096
Age (years)	0.856	0.994	0.937–1.056
Male	0.914	1.083	0.254–4.630
Hypertension	0.129	0.366	0.100–1.339
Diabetes	0.754	0.800	0.199–3.223
BMI (kg/m^2^)	0.663	0.945	0.734–1.218
LVEDD (mm)	0.117	0.929	0.847–1.019
LVESD (mm)	0.109	0.930	0.851–1.016
E/e'	0.736	1.017	0.921–1.123
E/A	0.115	0.441	0.159–1.220
LVEF (%)	0.274	0.957	0.885–1.035
Total volume EAT (ml)	0.690	0.997	0.985–1.010
Anterior wall (mm)	0.965	1.069	0.055–20.867
Inferior wall (mm)	0.944	0.847	0.008–89.034
Left free wall (mm)	0.411	0.195	0.004–9.641
Right free wall (mm)	0.127	0.090	0.004–1.973
Ventricle apex EAT	0.050	0.028	0.001–1.000
Right AV groove EAT	0.032	0.187	0.041–0.865
Left AV groove EAT	0.031	0.079	0.008–0.792
Scar burden	0.105	0.952	0.897–1.010
TPD	0.073	0.907	0.816–1.009
Systolic PSD	0.042	0.961	0.924–0.999
Systolic PBW	0.031	0.990	0.981–0.999

*CI, confidence interval; BMI, body mass index; NT-proBNP, N-terminal pro-natriuretic brain natriuretic peptide; ACE, angiotensin-converting enzyme; ARBs, angiotensin II receptor blocker; LVEDD, left ventricular end-diastolic dimension; LVESD, left ventricular end-systolic dimension; E/e', mitral inflow E velocity to tissue Doppler e' velocity ratio; E/A, mitral inflow E velocity to mitral inflow A velocity ratio; LVEF, left ventricular ejection fraction; TPD, total perfusion deficit; PSD, phase standard deviation; PBW, phase histogram bandwidth; AV, atrial ventricular; EAT, epicardial adipose tissue*.

**Table 3:** Multivariate logistic regression models for CRT response.

	**Wald**	***p*-value**	**Hazard Ratio** **(95% CI)**	**95% CI**
**Model 1-with-ventricle apex EAT**				
Dyssynchrony	0.092	0.762	0.779	0.155–3.913
QRS duration	4.762	0.029	1.051	1.005–1.100
TPD	0.931	0.335	0.947	0.848–1.058
Ventricle apex EAT	2.851	0.091	0.013	0.000–2.007
**Model 2-with right AV groove EAT**				
Dyssynchrony	0.188	0.664	1.431	0.284–7.219
QRS duration	2.594	0.107	1.037	0.992–1.084
TPD	3.184	0.074	0.898	0.799–1.011
Right AV groove EAT	4.075	0.044	0.156	0.026–0.948
**Model 3-with left AV groove EAT**				
Dyssynchrony	0.290	0.590	1.571	0.304–8.118
QRS duration	3.422	0.064	1.041	0.998–1.085
TPD	2.363	0.124	0.915	0.817–1.025
Left AV groove EAT	2.947	0.086	0.089	0.006–1.409

*TPD, total perfusion deficit; PSD, phase standard deviation; PBW, phase histogram bandwidth; AV, atrial ventricle; EAT, Epicardial adipose tissue*.

### Follow-Up After CRT

**Figure 4:**
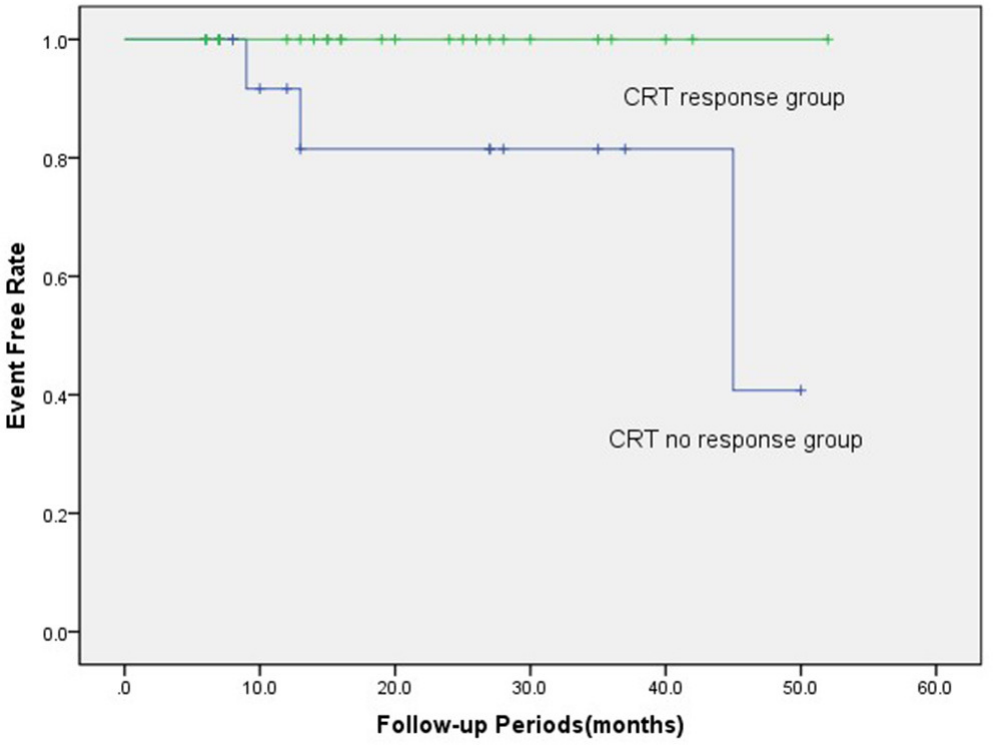
In the Kaplan-Meier survival analyses, the CRT response group had a better prognosis than the CRT non-response group in terms of all-cause mortality (log-rank χ^2^ = 3.971, *p* = 0.046). There were three (7.3%) patients who died from all-cause mortality, which all were included in the CRT non-response group. CRT, cardiac resynchronization therapy.

Over the entire period of 21.5 months (IQR 6–59 months), three (7.3%) patients died of all-cause mortality, and they were all were included in the CRT non-response group (*p* = 0.089). There was no significant difference in the number of new AF during follow-up in the response group and the no response group (2 vs. 3, *p* = 0.375). Meanwhile, the ventricular pacing rates were 94.5 and 91.6% in the response group and the no response group (*p* = 0.061), respectively. Meanwhile, the LVEF was significantly increased compared with the baseline (29.0 ± 8.5 vs. 40.6 ± 14.4%, *p* < 0.001). In the Kaplan-Meier survival analysis, the CRT response group had a better prognosis in the long term than the non-response group (log-rank χ^2^ = 3.971, *p* = 0.046; Figure 4).

## Discussion

The main findings of our study are as follows: (1) the EAT thickness of the left AV groove is associated with total defect perfusion of the left ventricle and LV systolic synchrony in patients with non-ischemic systolic HF; and (2) the EAT thickness of the AV groove is predictive of CRT response at 6 months in patients with non-ischemic systolic HF.

### The Clinical Explanation for the Lack of CRT Response

The CRT value has been widely evaluated in patients with HF in a large number of clinical trials. Despite moderate improvement over the past two decades, the CRT response is still rather mixed, and approximately one-third of patients with HF do not show benefits from CRT implantation. Therefore, it is imperative to enhance CRT benefits by patient selection, LV lead placement optimization, post-implantation device programs, and patient management. Numerous attempts have already been made to find reliable parameters to improve the CRT response. Different studies have reported the significance of LV myocardial scars in CRT non-response (12, 13). In addition, other studies have found that the CRT response was lower in patients with ischemic HF than in non-ischemic patients with HF because of the extensive burden on the left ventricle of scarred myocardium (14). Additionally, other researchers (15) reported that extensive scar burden in patients with CRT led to worse clinical outcomes and less improvement in LVEF. Compared with earlier findings, however, Riedlbauchova et al. (16) reported that the extent of scar burden was not predictive of CRT response or mortality, and they reported that the scar burden was higher than 40% in all groups, which appears to be much higher than that in a previous report (17, 18). In our study, a trend toward higher scar burdens was observed in the CRT non-response group, although the difference was not significant. In addition, the total deficit perfusion of the left ventricle in the CRT non-response group was lower than that in the CRT response group. However, the scar burden and TPD were not predictive of CRT response in our research. One explanation is that the higher myocardial scar burden might weaken the predictive value of these parameters in CRT (16).

In accordance with previous results, our study also confirmed that a wider QRS complex was a strong predictor of CRT response (19). When the EAT thickness parameters (LV apex, right AV groove, and left AV groove) were separately included in the multivariate logistic regression, a wider QRS complex remained an effective predictor of CRT implantation.

The use of LV dyssynchrony to predict benefits from CRT in patients with HF has been discussed in recent studies. Several recent studies found that LV mechanical dyssynchrony could predict CRT response during follow-up (20, 21). However, some reports have provided contradictory conclusions about the value of mechanical dyssynchrony in predicting CRT response, and they suggested that the baseline dyssynchrony parameters did not have a positive predictive value for CRT response in relatively large samples (22, 23). Nevertheless, our previous study found that baseline mechanical dyssynchrony from gated MPI was a significant independent predictive factor for CRT response in patients with non-ischemic systolic HF. In the present study, dyssynchrony parameters in both the PSD and PBW were significantly different in the CRT non-response groups. Meanwhile, the mechanical dyssynchrony parameters of both systolic PSD and systolic PBW were significantly associated with CRT response in the univariate logistic regression analysis. However, the mechanical dyssynchrony parameters were not significant independent predictors for CRT response in the multivariate logistic regression models. A reasonable explanation is that our population is relatively small compared with that in the previous study (3).

### Significance of the EAT in CRT

A novel finding of our work is that the EAT thickness of the AV groove has predictive value for CRT implementation in patients with non-ischemic systolic HF. This study is the first to relate the volumes and distribution of EAT to CRT response. In a previous study, EAT was found to be a directly adjacent tissue to the cardiac myocardium, which is a vital regulator of the energy needs of the heart myocardium in lipid fluxes (24). However, expanding EAT becomes hypoxic and dysfunctional in cardiovascular disease related to metabolic processes, which would cause a reduction in protective cytokines, accumulation of detrimental adipocytokines, and extensive fibrosis in the myocardium (25). Up to now, the correlation of EAT with LV function is still inconsistent (4, 26, 27). A meta-analysis reported that EAT is associated with diastolic function, independent of other influential variables (28). While EAT is an effect modifier for chamber size but not systolic function. Meanwhile, van Woerden (29) revealed that the volume of EAT in patients with HF was larger than that in the controls despite a similar BMI in recent years. Furthermore, the EAT volume was associated with AF, type 2 diabetes mellitus, and myocardial injury-related biomarkers. Wu et al. (4) concluded that EAT was closely linked with the extent of myocardial fibrosis and heart dysfunction in the pathophysiology of HF. Moreover, Maimaituxun et al. (30) found that localized EAT (AV groove and left free wall) was strongly associated with LV function in preserved patients with LVEF. In our results, the EAT thickness of the left AV groove was associated with total defect perfusion of the left ventricle as measured by SPECT, and it was influenced by increases in the severity of ischemia or global fibrosis (31, 32). We further investigated the parameters of the whole heart (total volume EAT) and localized EAT (left anterior wall, inferior wall, left free wall, right free wall, apex, right AV groove, and left AV groove) in patients with non-ischemic systolic HF. The total volume EAT was not associated with LVEF, and LV scar burden in patients with non-ischemic systolic HF and the volume of EAT was similar between the two groups. However, the thickness of the localized EAT (LV apex, right AV groove, and left AV groove) was higher in the non-response group. This conclusion is also consistent with a previous study which concluded that regional EAT is associated with alterations in local cardiac structure and function (33). More importantly, the right AV groove and left AV groove were both independent predictors of CRT response. This finding suggested that localized EAT in the right AV groove and left AV groove is related to cardiac resynchronization and thus to the CRT response. Furthermore, the strong link between localized EAT in the left AV groove and systolic LVMD (SD and BW) further clarified that evaluation of localized EAT would alter the dilemma of CRT response.

Nevertheless, the potential mechanisms between EAT and CRT response remain to be elucidated, which might partly be attributed to mechanical and paracrine processes. The physical boundary in anatomy between the EAT and ventricular myocardium is not obvious and shares the same coronary microcirculation. Therefore, factors contributed by EAT could have direct vasocrine and paracrine effects on the ventricular myocardium, which worsen myocardial fibrosis by localized EAT accumulation (34). Previous studies on EAT may explain its value in predicting CRT response. Reasonable speculation is that EAT accumulation affects myocardial fibrosis, which influences CRT response [35], especially in the LV lead position (lateral wall or posterior lateral wall). Meanwhile, localized EAT accumulation around the right AV groove and left AV groove is directly related to impaired motion (30), such as, mechanical dyssynchrony, which was widely accepted corresponding to CRT response. On the other hand, EAT is a relevant electrophysiological factor in CRT patients that may interfere with pacing and sensing (7). Meanwhile, greater EAT thickness might increase under LV lead sensing and reduce battery longevity. Therefore, the evaluation of EAT might be a novel factor for predicting the CRT response rate and selecting patients for CRT implantation. Furthermore, EAT has the potential for therapeutic use in sodium dependent glucose transporters 2 (SGLT-2) inhibitor treatment to improve the CRT response rate.

## Limitation

First, the samples in our retrospective study were relatively small, which was a disadvantage for identifying the predictive factors for CRT response in HF. While, the present retrospective study included patients with non-ischemic systolic HF, undergoing CRT therapy, and receiving ECT and contrast-enhanced cardiac CT scans. So far, there was limited data about the association between EATs and CRT response. Second, the definition of CRT non-response was based on echocardiographic results of LVEF to measure the primary end point. We did not investigate other clinical factors, such as the 6-min walking test or quality of life. Third, the follow-up period was rather short for post-CRT implantation in the current research. Fourth, EAT parameters were limited in all patients with CRT, and our sample was not compared with an adequate control group. Finally, this study was still preliminary, and further research should be undertaken in a larger population.

## Conclusion

The EAT thickness of the left AV groove is associated with total defect perfusion of the left ventricle and LV systolic synchrony in patients with non-ischemic systolic HF. The EAT thickness of the AV groove has predictive value for CRT response in patients with non-ischemic systolic HF.

## Data Availability Statement

The raw data supporting the conclusions of this article will be made available by the authors, without undue reservation.

## Ethics Statement

The studies involving human participants were reviewed and approved by Institutional Ethical Committee of the First Affiliated Hospital of Nanjing Medical University. The patients/participants provided their written informed consent to participate in this study.

## Author Contributions

H-yQ and CW performed the clinical study and drafted the original manuscript. H-yQ and D-dQ enrolled the patients and analyzed all of the clinical data. CC and M-lC reviewed and edited the manuscript. All authors contributed to the whole article and approved the final version of this manuscript.

## Funding

This research was supported by grants from the National Nature Science Foundation of China (81900295 and 82100338) and the Natural Science Foundation of Jiangsu Province (BK 20191071).

## Conflict of Interest

The authors declare that the research was conducted in the absence of any commercial or financial relationships that could be construed as a potential conflict of interest.

## Publisher's Note

All claims expressed in this article are solely those of the authors and do not necessarily represent those of their affiliated organizations, or those of the publisher, the editors and the reviewers. Any product that may be evaluated in this article, or claim that may be made by its manufacturer, is not guaranteed or endorsed by the publisher.
